# Deletion of the forebrain mineralocorticoid receptor impairs social discrimination and decision-making in male, but not in female mice

**DOI:** 10.3389/fnbeh.2014.00026

**Published:** 2014-02-06

**Authors:** Judith P. ter Horst, Maaike van der Mark, Jiska Kentrop, Marit Arp, Rixt van der Veen, E. Ronald de Kloet, Melly S. Oitzl

**Affiliations:** ^1^Swammerdam Institute for Life Sciences, Center for Neuroscience, University of AmsterdamAmsterdam, Netherlands; ^2^Department of Clinical Psychology, University of AmsterdamAmsterdam, Netherlands; ^3^Department of Urology, LUMC, Leiden UniversityLeiden, Netherlands; ^4^Department of Endocrinology, Leiden University Medical Center & Medical Pharmacology, Leiden Academic Center for Drug Research, Leiden UniversityLeiden, Netherlands; ^5^Centre for Child and Family Studies, Leiden UniversityLeiden, Netherlands

**Keywords:** mineralocorticoid receptor, knockout mice, social approach, social discrimination, sex

## Abstract

Social interaction with unknown individuals requires fast processing of information to decide whether it is friend or foe. This process of discrimination and decision-making is stressful and triggers secretion of corticosterone activating mineralocorticoid receptor (MR) and glucocorticoid receptor (GR). The MR is involved in appraisal of novel experiences and risk assessment. Recently, we have demonstrated in a dual-solution memory task that MR plays a role in the early stage of information processing and decision-making. Here we examined social approach and social discrimination in male and female mice lacking MR from hippocampal-amygdala-prefrontal circuitry and controls. The social approach task allows the assessment of time spent with an unfamiliar mouse and the ability to discriminate between familiar and unfamiliar conspecifics. The male and female test mice were both more interested in the social than the non-social experience and deletion of their limbic MR increased the time spent with an unfamiliar mouse. Unlike controls, the male MR^CaMKCre^ mice were not able to discriminate between an unfamiliar and the familiar mouse. However, the female MR mutant had retained the discriminative ability between unfamiliar and familiar mice. Administration of the MR antagonist RU28318 to male mice supported the role of the MR in the discrimination between an unfamiliar mouse and a non-social stimulus. No effect was found with a GR antagonist. Our findings suggest that MR is involved in sociability and social discrimination in a sex-specific manner through inhibitory control exerted putatively via limbic-hippocampal efferents. The ability to discriminate between familiar and unfamiliar conspecifics is of uttermost importance for territorial defense and depends on a role of MR in decision-making.

## Introduction

Discriminating social from non-social stimuli and recognizing whether they are “familiar” or “unknown” is an essential prerequisite for adequate responses in social contacts. In such social interactions with unknown conspecifics fast processing of information is required to decide if this new individual is a friend or foe. During this interaction the behavioral response will be approach, exploration, anticipation, fight or avoidance (e.g., freeze or flight away from the conspecific). This is a stressful situation with impact on the hierarchy in social groups and ultimately on survival. Indeed, both social defeat and victory cause a rise in the activity of the hypothalamic-pituitary-adrenal (HPA) axis and corticosterone, although the duration of corticosterone secretion is prolonged in the defeated animal (Koolhaas et al., 2011).

Corticosterone binds to the mineralocorticoid (MR) and glucocorticoid receptor (GR) in the brain. The affinity of the MR to corticosterone is tenfold higher than that of the GR. The MR is abundantly expressed in limbic-cortical areas, particularly in neurons of hippocampus, dorsolateral septum, amygdala, indusium griseum and some cortical areas among which the olfactory tubercle, anterior olfactory nucleus and layer III of the pyriform cortex (Ahima et al., 1991). In these neurons MR is co-localized with GR, which occurs throughout the brain. The slow genomic effect of corticosterone is mediated via nuclear MR and GR (Joels, 1997, 2007). Recently, a fast non-genomic mode of action of corticosterone involving membrane MR and GR was discovered (Di et al., 2003; Karst et al., 2005, 2010; Groeneweg et al., 2011).

In response to corticosterone activation, both MR and GR play a role in emotion and cognition (de Kloet et al., 2005). The MR is involved in the appraisal and risk assessment in novel situations (Oitzl and de Kloet, 1992; Lupien and Mcewen, 1997) indicating its role in the early part of information processing and decision-making. Via GR, newly learned information is consolidated in the memory for future use when a similar situation is encountered (Oitzl and de Kloet, 1992; Roozendaal et al., 1996; Lupien and Mcewen, 1997; Oitzl et al., 2010). The necessity for a balance between MR and GR is shown by fludrocortisone in an aversive olfactory fear conditioning (OFC) task where risk assessment and consolidation of the aversive experience is promoted depending on the time of administration of this mixed MR and GR agonist (Souza et al., 2013). Furthermore, in mice with forebrain MR over-expression and/or simultaneous global GR under-expression the stress-induced HPA axis activation was attenuated, while perseveration of fear-motivated behavior was enhanced especially with the higher MR:GR ratio (Harris et al., 2013). Collectively, these studies demonstrate that a balance of MR and GR is crucial for behavioral adaptation to stress (de Kloet et al., 1998, 2005).

In this study, we test the hypothesis that the limbic MR is involved in social approach behavior and social discrimination. Previous studies showed that adrenalectomized rats lacking corticosterone had very low levels of social interaction which could be normalized by corticosterone replacement sufficient to occupy MR (File et al., 1979). In addition, glucocorticoid deficiency caused by metyrapone administration resulted in increased aggressive attacks and reduced social interaction (Haller et al., 2004). However, acute blockade of the MR inhibited aggressive behavior in male rats (Haller et al., 1998, 2000) and prevents the propensity of aggressive behavior (Kruk et al., 2013). Collectively, these studies demonstrate an intriguing complexity in the regulation of social contact.

To test our hypothesis we raise the following questions: does deletion of the limbic MR affect the ability to discriminate (i) between social and non-social stimuli; (ii) between familiar and unfamiliar conspecifics?; and (iii) is there a sex difference in the effect of MR deletion from the limbic circuitry? The “social approach task” allows testing these two discriminative abilities but avoids an aggressive behavioral response (Moy et al., 2004; Nadler et al., 2004; Page et al., 2009). Generally, mice prefer to explore conspecifics over objects (File and Hyde, 1978) and will show more interest in unfamiliar than familiar conspecifics (Young, 2002; Toth and Neumann, 2013).

In our experimental approach we use MR^CaMKCre^ mice in which the MR is genetically deleted from hippocampus, lateral septum and amygdala after the first postnatal days (Berger et al., 2006). These areas express the highest density of MR (Ahima et al., 1991) and constitute part of the limbic circuitry with efferent projections to frontal cortical brain regions. Loss of limbic MR in MR^CaMKCre^ mice appeared to affect behavioral flexibility (Berger et al., 2006) as expressed by delayed learning of the water-maze and the circular hole board task and deficits in working memory on the radial maze (Berger et al., 2006; ter Horst et al., 2012b, 2013a). Furthermore, MR^CaMKCre^ mice showed hyperreactivity towards a novel object and increased arousal under stress (Berger et al., 2006; Brinks et al., 2009). We also observed remarkable sex-differences: male MR^CaMKCre^ and control mice were able to extinguish their fear and to discriminate between “safe” and “threat” episodes of a fear conditioning task, whereas female MR^CaMKCre^ mice could not (Brinks et al., 2009; ter Horst et al., 2012a). Given the increased response of MR^CaMKCre^ towards a novel object (Berger et al., 2006), we expected a hyper-responsive reaction towards an unknown mouse.

Here we demonstrate that male and female test mice show increased attention to the social rather than non-social stimuli and that sociability is enhanced following genetic deletion of the MR from hippocampus, amygdala and septum. However, a sex difference emerged when the discriminative ability of the mutants is tested. Female, but not male, mutants retained the ability to discriminate between familiar and unfamiliar conspecifics. Subsequently, we showed that treatment of control littermates with a MR antagonist for acute blockade of the MR supported the evidence produced after genetic deletion of this receptor postnatally in social approach, discrimination and decision-making.

## Materials and methods

### Animals

Male and female MR^CaMKCre^ mice (5–8 months) and their control (MR^flox/flox^) littermates were bred in the animal facility of Leiden University. The MR^CaMKCre^ mice and their control males were obtained by breeding MR^flox/flox^ with MR^flox/wtCaMKCre^ mice (kindly given by the German Cancer Research Center, Heidelberg, Germany). A modified MR allele (MR^flox^) was generated in embryonic stem cells of 129Ola mice and the CaMKCre transgene was injected in FVB/N mice (Casanova et al., 2001). The MR^flox^ allele and CaMKCre transgene were backcrossed into C57BL/6J for multiple generations. This resulted in a loss of MR protein expression in the lateral septum, indusium griseum, the *cornu ammonis* (CA) and dentate gyrus regions of the hippocampus and amygdala (Berger et al., 2006).

Furthermore, same sex C57BL/6J mice (*n* = 8 per experiment, female strangers for female MR^CaMKCre^ mice and male strangers for male MR^CaMKCre^ mice; 4 months old; purchased from Janvier, France) were used as “stranger” mice in the sociability task. These C57BL/6J mice were housed in a separate room to avoid any contact with the other mice. One week before the experiment the MR^CaMKCre^ and MR^flox/flox^ control mice were moved to the experiment room and housed individually in Macrolon cages (translucent plastic: 44 × 22 × 17 cm) with sawdust bedding, a tissue for nest building, water and food *ad libitum*, at 20°C with controlled humidity under a 12:12 h light/dark cycle (with lights on at 7.30 h). Experiments were performed between 08.30 and 12.30 h, approved by the committee on Animal Health and Care from Leiden University, The Netherlands, in accordance with the EC Council Directive of September 2010 (2010/63/EU).

### Social approach

The task consists of three consecutive parts: habituation, sociability and social discrimination. MR^CaMKCre^ (male: *n* = 7–8; female: *n* = 16) and MR^flox/flox^ control mice (male: *n* = 7–9; female: *n* = 15) will be referred to as “test” mice, while C57BL/6J will be called “stranger” mice.

#### Apparatus

**Figure 1:**
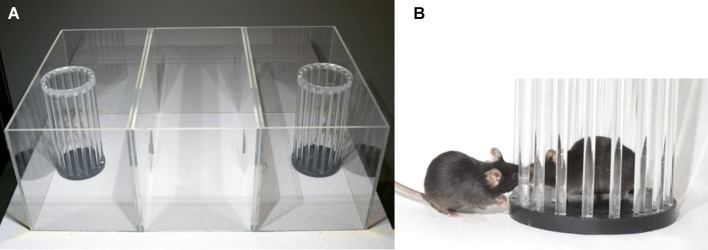
**Social approach apparatus. (A)** A picture of the apparatus containing the two cylinders; **(B)** close-up of an interaction between the stranger mouse in the cylinder and the test mouse.

The apparatus is made of transparent plexiglass walls (Figure 1, Noldus Information and Technology BV, Wageningen, The Netherlands). The box measures 59 × 39.5 × 21.5 cm and is divided into three chambers of equal size (18.5 × 39.5 cm) by walls with a 7 × 7 cm square opening that could be closed by a slide door. Each of the two side chambers contains a cylinder. These cylinders (20 × 10 cm diameter) are made out of 18 transparent plexiglass bars placed 6 mm apart; the upper end of the cylinder is closed with a black lid. At the end of the 20 min task, all mice return to their home cage. The apparatus is cleaned with tap water and 70% EtOH and dried.

#### Stranger mice

Adult male and female C57BL/6J mice used as the social stimulus are called “stranger mouse”. Female strangers were used for female MR^CaMKCre^ and control mice and male strangers for male MR^CaMKCre^ mice and controls. Strangers are housed two per cage in a separate room to avoid visual, auditory, and olfactory contact with the test mice. One day before the start of testing, the strangers are placed into the cylinders in the social test apparatus for 5 min. This should allow some familiarization with the environment. Each stranger is used twice per day. The stranger for the sociability test and the stranger for the preference for social novelty test are taken from different cages. Keeping the stranger mouse in a cylinder prevents aggressive and sexual interactions, also ensuring that all social approach is initiated by the test mouse. Experiments from Nadler et al. (2004) have indicated that the strain of the stranger does not change the social approach of the test mouse.

#### Behavioral parameters

Sessions are video-taped and analyzed with Ethovision XT 6.1 (Noldus Information and Technology BV, Wageningen, The Netherlands). The location and movement of the test mouse is analyzed using the following parameters for general activity: distance and velocity; preference to be at a certain location: time spent (in seconds and percentage) in middle and side chambers; time spent and entries into a defined zone around the cylinders (1.5 × the size of the cylinder).

#### Experimental procedure

The experimental procedure is adapted from (Moy et al., 2007) and (Page et al., 2009), and consists of three consecutive parts:

1. Habituation (5 min): The test mouse is placed in the middle chamber, sliding doors are opened and the mouse is allowed to explore all three chambers. The two side chambers contain an empty cylinder. The empty cylinder presents a novel inanimate object without social value. General activity, possible preference for a certain part of the apparatus (middle or one of the side chambers) and exploration of the cylinder are measured.
2. Sociability (10 min): After the habituation period, the sliding doors are shut and the test mouse is enclosed in the middle chamber. An unfamiliar mouse (stranger) is put into one of the cylinders and placed in one of the side chambers. The location for stranger is alternated, either to the left or right chamber of the social test box. The cylinder in the other chamber is empty. Following placement of stranger, the doors are opened, and the test mouse has access to all three chambers. Increased time spent in the chamber and in the perimeter around the cylinder with the stranger indicates preference for the social stimulus compared to the empty cage. For extra analysis, this 10 min period is also subdivided into two blocks of 5 min.
3. Social discrimination (5 min): Again, the test mouse is confined to the middle chamber. Another unfamiliar mouse (new stranger) is placed in the cylinder that was empty during the sociability test. The “old” stranger remains in position in its cylinder and chamber. The sliding doors are opened and the test mouse gets access to the side chambers. It is expected that the test mouse will spent more time with the new stranger than the old stranger. Increased time spent with the new stranger is a measure for the discriminative ability of the test mouse, also indicative for intact working memory.

##### Experiment 1: social approach behavior of male MR^CaMKCre^ mice

Naïve male MR^CaMKCre^ (*n* = 8) and control mice (*n* = 9) were tested for their response in the social approach paradigm.

##### Experiment 2: social approach behavior of female MR^CaMKCre^ mice

Naive female MR^CaMKCre^ (*n* = 16) and control mice (*n* = 15) were tested for their response in the social approach paradigm. To take the estrous cycle into account we used twice the amount of females than males. To test for the estrous cycle: smears were taken after the social approach (ter Horst et al., 2013b). The mouse was placed on top of its cage, lifted slightly by its tail and the head of the smear loop (size 1 μl; Greiner Bio-one) was gently inserted above the major labia in the cloaca and carefully rubbed along the ventral/rostral side of the cloaca. Cells were transferred to a drop of water on a glass microscope slide, air-dried and stained with Giemsa (Sigma) to facilitate identification of the cycle phase. The four phases are proestrus, estrus, metestrus and diestrus. We did not encounter enough females (test and stranger mice) in each phase of the estrous cycle to be able to perform a statistical analysis and therefore did not include it in this study.

##### Experiment 3: receptor type involved in social approach behavior in male mice

To determine the role of the corticosteroid receptors in the social approach behavior, we treated control male mice with either a MR antagonist or a GR antagonist. Mice (*n* = 8) received the GR antagonist RU38486 (Mifepristone, also known as a progesterone antagonist; Sigma; 10 mg/kg of body weight; Zhou et al., 2010) intraperitoneally 45 min before the start of the social approach test. RU38486 was dissolved in 0.9% NaCl containing 0.25% carboxymethylcellulose and 0.2% Tween-20. Solutions were prepared on the day before the injection and vortexed overnight.

A second group of mice (*n* = 8) received the MR antagonist RU28318 (*Tocris Bioscience*; 50 mg/kg body weight; dissolved in physiological saline (Schwabe et al., 2010)) subcutaneously 45 min before the start of the social approach test. Solutions were prepared on the day before the injection, frozen, and defrosted 30 min before injection.

After the injection, mice returned to their home cage and the social approach test started 45 min later. These mice were tested in the habituation and sociability phase only. One week later the mice which previously had received RU28318 were tested again with new strangers in the sociability phase.

### Buried pellet test

A pilot study was conducted to examine the ability to smell in male MR^CaMKCre^ and control mice. Two days before testing, mice (*n* = 6) were housed individually and received 3 raisins (1.3 g) and 1 food pellet (1.3 g) per mouse with water *ad libitum* each day. It was noted on day 2 that the mice preferred the food pellet instead of the raisins but did eat both. At the day of testing, mice were placed individually into a clear Macrolon cage (translucent plastic: 44 × 22 × 17 cm) in which 1 food pellet of 1.3 g was hidden under a 1.5 cm layer of standard sawdust bedding at the far end of the cage. The mouse was placed into the box opposite to the location of the pellet and allowed to explore for 5 min. The latency to retrieve the hidden food pellet was recorded. All mice, but one, found and ate the hidden food pellet within 5 min. These results show that the mice are able to smell the food. The one mouse which did not find the food pellet was very actively moving and exploring the cage but was not further included in the study.

### Corticosterone measurements

Blood samples were obtained by a small incision at the base of the tail (Dalm et al., 2008), plasma was isolated and corticosterone concentrations were measured using a commercially available radio immune assay (MP Biomedicals Inc. Europe, Belgium; sensitivity 3 ng/ml). Blood samples reflecting basal resting levels of corticosterone were taken in the week before the experiment started, between 9.00 and 10.00 h. Immediately after the social approach task blood samples were taken as well.

### Statistical analysis

A MANOVA was used to test for main significant effects of genotype, treatment and/or time per chamber followed by within-group *post hoc* LSD comparisons. For general activity and perimeter analysis an unpaired two-tailed Student’s *t*-test was used. Statistical significance of all tests was determined as *p* < 0.05. All data are expressed as mean ± S.E.M.

## Results

### General activity and habituation

**Figure 2:**
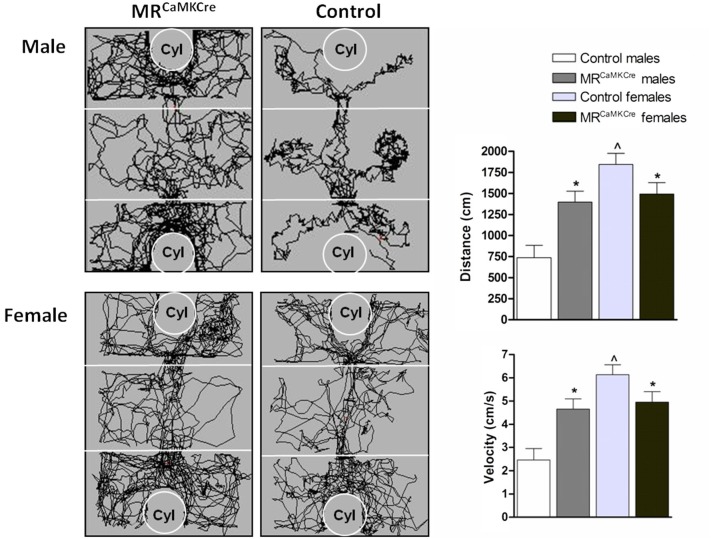
**Walking pattern, distance walked and velocity during habituation**. Male MR^CaMKCre^ mice showed increased locomotion compared to control littermates. Female MR^CaMKCre^ showed decreased locomotor activity compared to their control littermates. White bars represent control males (*n* = 9) and light grey bars represent the female control mice (*n* = 15). Dark grey represent the male MR^CaMKCre^ mice (*n* = 8) and black bars the female MR^CaMKCre^ mice. * *p* < 0.05 compared to controls; *p* < 0.05 compared to males. All data are expressed as mean ± S.E.M. Cyl represents cylinder.

*General activity*: Male MR^CaMKCre^ mice traveled longer distance and had a higher velocity than controls over all test periods (distance: *F*_(1,15)_ = 13.133, *p* < 0.01; velocity *F*_(1,15)_ = 13.154, *p* < 0.01; Figure 2). In female mice, the controls moved more and faster than MR^CaMKCre^ mice over all test periods (distance: *F*_(1,29)_ = 6.616, *p* < 0.05; velocity: *F*_(1,29)_ = 6.618, *p* < 0.05). Distance walked and velocity was comparable in male and female MR^CaMKCre^ mice. However, control females were significantly more active than control males (*p* < 0.0001; Figure 2).

*Habituation*: Male MR^CaMKCre^ mice were more active in the exploration of their environment and distributed their activity more evenly over the three chambers than the control littermates who preferred staying in the middle chamber (genotype × chamber: *F*_(2,45)_ = 4.478, *p* < 0.05, Figure 2). Female mice (MR^CaMKCre^ and controls) explored all three chambers evenly (genotype × chamber: *F*_(2,87)_ = 0.344, *p* < 0.1). No differences in latency to leave the middle chamber were found between MR^CaMKCre^ and control mice (6.2 ± 1.8 vs. 7.6 ± 2.2, respectively).

*Sex differences*: Male and female MR^CaMKCre^ mice showed comparable behavior during habituation; while male and female control mice differed significantly (MR^CaMKCre^ gender × chamber: *F*_(2,66)_ = 0.810, *p* < 0.1; controls gender × chamber: *F*_(2,66)_ = 10.617, *p* < 0.0001). Compared to male controls, female mice left the middle chamber significantly faster (*p* < 0.05; data not shown).

### Sociability

**Figure 3:**
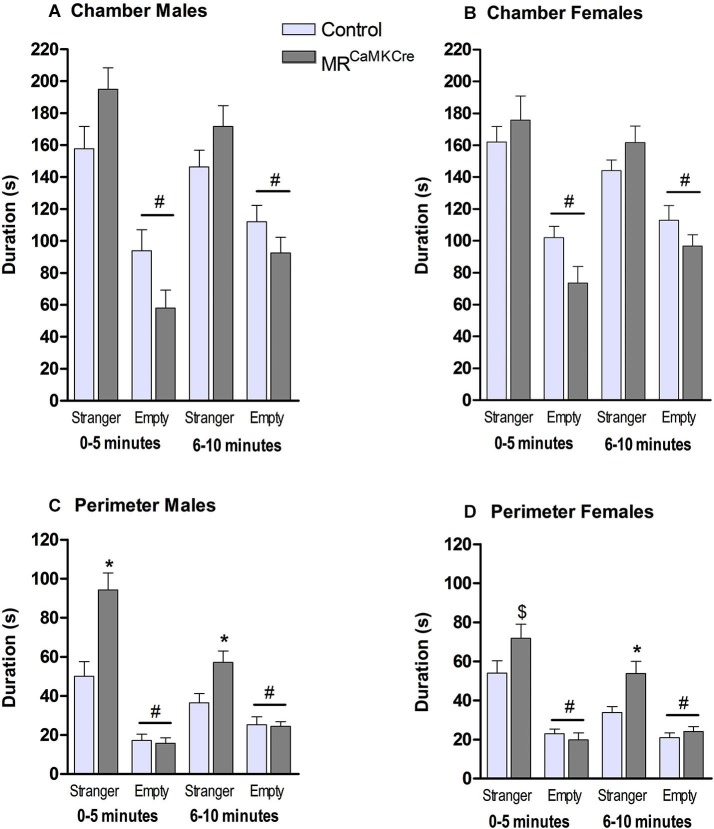
**Sociability in male and female MR^CaMKCre^ and control mice.** Time in seconds, spent in **(A)** the chamber by males and **(B)** the chamber by females; **(C)** the perimeter around the cylinder (males); **(D)** the perimeter around the cylinder (females). Light grey bars represent the control mice (*n* = 9 (males); *n* = 15 (females)) and dark grey the MR^CaMKCre^ mice (*n* = 8 (males); *n* = 16 (females)). * *p* < 0.05 compared to controls same treatment; ^#^ *p* < 0.05 compared to the stranger chamber/perimeter. ^$^ *p* < 0.05 compared to males. All data are expressed as mean ± S.E.M.

*Male*: Both genotypes stayed significantly longer in the chamber with the stranger than in the chamber with the empty cylinder (*p* < 0.001) which was indicative for social approach towards the stranger mouse. During the 10 min, MR^CaMKCre^ mice spent more time in the chamber with the stranger than controls (*F*_(1,15)_ = 6.303, *p* < 0.05). Dividing the time spent in the chamber in two blocks of 5 min showed a statistical trend (Figure 3A; 0–5 min: *p* < 0.1; 6–10 min: *p* < 0.1). Calculating the time spent in the perimeter around the cylinders revealed that MR^CaMKCre^ mice spent significantly more time with the stranger than controls (total 10 min: *F*_(1,15)_ = 16.427, *p* < 0.01; 0–5 min: *p* < 0.01; 6–10 min: *p* < 0.05). During the first 5 min, both MR^CaMKCre^ and control mice spent more time in the perimeter of the stranger than near the empty cylinder (Figure 3C, *p* < 0.001), however during the second 5 min only the MR^CaMKCre^ mice stayed longer with the stranger (MR^CaMKCre^: *p* < 0.01 and control: *p* < 0.1). Overall, this indicated that the time spent in the chamber was indeed dedicated to approach and investigation of the stranger mouse. Time spent in the middle chamber was about 20–40 s and comparable between genotypes.

*Female*: Both MR^CaMKCre^ and control mice were more interested in the chamber with the female stranger than the empty chamber (*p* < 0.0001). During the 10 min both genotypes spent a comparable amount of time in the chamber with the stranger (*F*_(1.29)_ = 1.403, *p* > 0.1, Figure 3B). During the 10 min MR^CaMKCre^ mice stayed significantly longer in the perimeter of the cylinder with the stranger than controls did (*F*_(1,29)_ = 7.579, *p* < 0.05, Figure 3D) but this was mainly due to the second 5 min (*p* < 0.01). Both genotypes spend more time in the perimeter of the cylinder with the stranger than of the empty cylinder. Time spent in the middle chamber was about 35–50 s and comparable between genotypes.

*Sex differences*: Male MR^CaMKCre^ spent more time in the proximity of the stranger than females did (*p* < 0.05).

### Social discrimination

**Figure 4:**
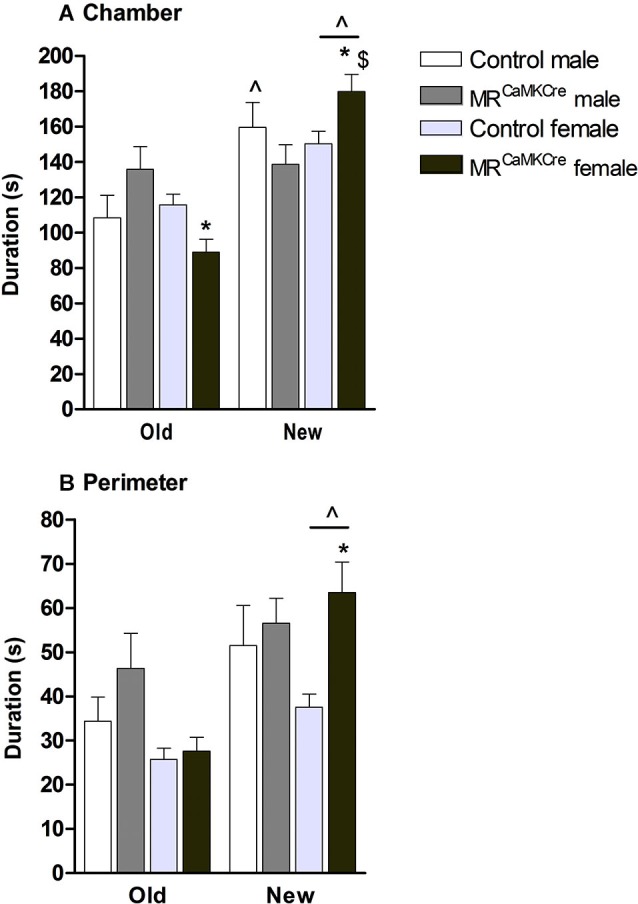
**Social discrimination in male and female MR^CaMKCre^ and control mice.** Time in seconds, spent in **(A)** the chamber and **(B)** the perimeter around the cylinder. White bars represent control males (*n* = 9) and light grey bars represent the female control mice (*n* = 15). Dark grey represent the male MR^CaMKCre^ mice (*n* = 8) and black bars the female MR^CaMKCre^ mice. * *p* < 0.05 compared to controls same treatment; ^$^ *p* < 0.05 compared to the males. ˆ *p* < 0.05 compared to old stranger. All data are expressed as mean ± S.E.M.

*Male*: Introducing another mouse, the “new stranger”, into the previously empty cylinder was expected to change the approach behavior towards the old stranger. Indeed, controls spent more time in the chamber of the new stranger than the old stranger (*p* < 0.01, Figure 4A) but no differences were found in the perimeter (*p* > 0.1, Figure 4B). MR^CaMKCre^ mice spent an equal amount of time with both strangers.

*Female*: Both control and MR^CaMKCre^ mice spent more time with the new stranger compared to the old stranger (chamber: *p* < 0.001; perimeter: *p* < 0.005, Figure 4). Interestingly, MR^CaMKCre^ mice were even more interested in the new stranger than controls were (chamber: *p* < 0.05; perimeter: *p* < 0.01).

*Sex differences*: Female MR^CaMKCre^ mice spent more time in the chamber with the new stranger than male MR^CaMKCre^ mice did (*p* < 0.05).

### Corticosterone levels

**Figure 5:**
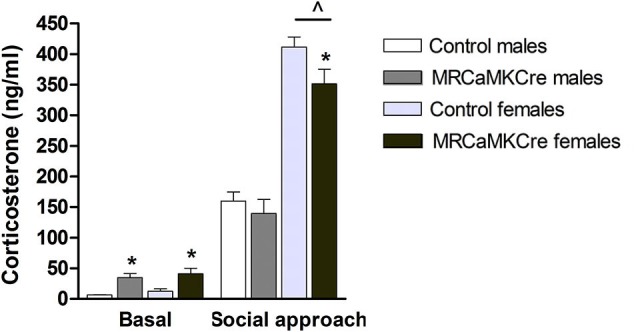
**Plasma corticosterone concentrations of male and female MR^CaMKCre^ and control mice**. Measured before and after the social approach paradigm. White bars represent control males (*n* = 9) and light grey bars represent the female control mice (*n* = 15). Dark grey represent the male MR^CaMKCre^ mice (*n* = 8) and black bars the female MR^CaMKCre^ mice. * *p* < 0.05 compared to controls same treatment; ˆ *p* < 0.05 compared to males. All data are expressed as mean ± S.E.M.

Basal plasma corticosterone concentrations were higher in male and female MR^CaMKCre^ mice compared to their control littermates (male: *t*_(13)_ = 4.116, *p* < 0.01; female: *t*_(35)_ = 2.838, *p* < 0.01; Figure 5). Social approach increased corticosterone: levels were comparable between male MR^CaMKCre^ mice and controls whereas female MR^CaMKCre^ mice had lower levels than control mice (*t*_(25)_ = 2.029, *p* = 0.05). This implies that meeting new conspecifics is stressful for both sexes but even more for females.

*Sex differences*: Basal corticosterone concentrations were comparable between male and female mice, depending on their genotype (controls: *t*_(24)_ = 1.462, *p* > 0.1; MR^CaMKCre^: *t*_(24)_ = 0.563, *p* > 0.1). After the social approach test the corticosterone levels of female mice, control and MR^CaMKCre^, were significantly increased compared to males (control: *t*_(20)_ = 10.559; *p* < 0.0001; MR^CaMKCre^: *t*_(19)_ = 5.652; *p* < 0.0001).

### Effect of mineralocorticoid receptor (MR) and glucocorticoid receptor (GR) antagonists on social approach behavior in male mice

In experiment 1, we observed that male MR^CaMKCre^ mice did not differentiate between the old stranger and the new stranger. To support the role of MR in this social paradigm we treated male control mice with either a MR antagonist in comparison with a GR antagonist.

**Figure 6:**
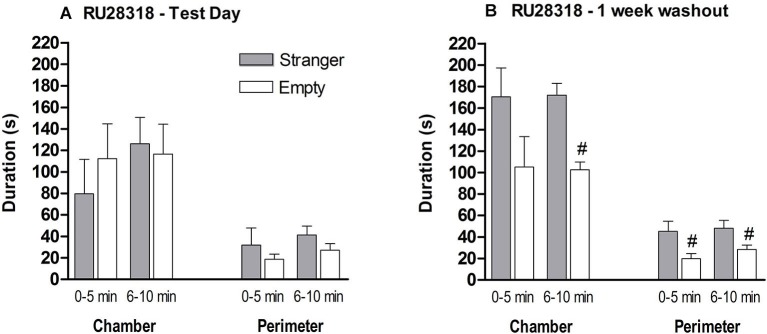
**The effect of MR (RU28318) antagonist on the sociability phase in male control mice. (A)** Time in seconds, spent in the chamber with the stranger mouse (grey bar) and in the chamber with the empty cylinder (white bar) for total time in chamber and time in the perimeter around the cylinder. **(B)** One week later the mice were tested again on time spent in the chamber and perimeter with a new set of strangers. ^#^ *p* < 0.05 compared to the stranger chamber/perimeter. All data are expressed as mean ± S.E.M.

*MR antagonist*: Behavior during the habituation was comparable between the naive and MR antagonist treated mice (data not shown). A stranger in one of the chambers did not influence the preference of chamber exploration. MR antagonist treated mice did not differentiate between the three chambers during the 10 min (*F*_(2,21)_ = 0.414, *p* > 0.1; Figure 6A). During the first 5 min, the mice spent around 80–120 s in middle chamber which decreased in the second 5 min block. During both 5 min blocks, time spent in the perimeter of the stranger and empty cylinder did not differ (0–5 min: *t*_(14)_ = 0.803, *p* > 0.1; 6–10 min: *t*_(14)_ = 1.374, *p* > 0.1). Since the treated mice could not differentiate between an unfamiliar mouse and an empty cylinder, we did not continue to test for social discrimination between familiar and unfamiliar mice.

One week after treatment, when RU28318 is not in their system anymore, the mice were re-tested for their sociability with a new stranger to demonstrate that the MR antagonist was responsible for the deficient sociability and not the mouse itself. Now they spent more time in the chamber with the stranger than in the empty one (0–5 min: *t*_(14)_ = 1.674, *p* > 0.1, 6–10 min: *t*_(14)_ = 5.291, *p* < 0.0001; Figure 6B). In addition, mice spent significantly more time during both 5 min blocks in the perimeter of the cylinder with the stranger than the empty cylinder (0–5 min: *t*_(14)_ = 2.438, *p* < 0.05; 6–10 min *t*_(14)_ = 2.333, *p* < 0.05).

**Figure 7:**
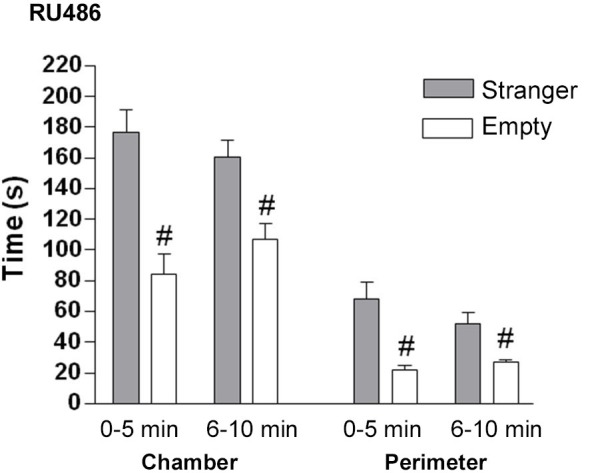
**The effect of GR (RU486) antagonist on the sociability phase in male control mice**. Time in seconds, spent in the chamber with the stranger mouse (grey bar) and in the chamber with the empty cylinder (white bar) for total time in chamber and time in the perimeter around the cylinder. ^#^ *p* < 0.05 compared to the stranger chamber/perimeter. All data are expressed as mean ± S.E.M.

*GR antagonist*: In the sociability phase, control mice treated with GR antagonist spent significantly more time in the chamber with the stranger than in the empty chamber (*F*_(2.21)_ = 59.4, *p* < 0.001 (Figure 7). GR antagonist treated mice spent significantly more time in the perimeter of the cylinder with the stranger mouse than in the empty cylinder (0–5 min: *t*_(14)_ = 3.97, *p* < 0.01; 6–10 min: *t*_(14)_ = 3.235, *p* < 0.05).

## Discussion

Here, we demonstrate that male mice without limbic MR (MR^CaMKCre^) and mice with pharmacological blockade of MR cannot discriminate between a familiar and an unfamiliar mouse, while male control mice prefer to spend time in the proximity of the unfamiliar mouse. This inability to discriminate is sex-specific, because female MR^CaMKCre^ mice, like their controls, spent more time close to the unfamiliar than the familiar mouse. All mice prefer the mouse over an object. Time spent in the proximity of an unfamiliar conspecific vs. an object was even higher in male and female MR^CaMKCre^ mice than in control mice. In contrast, pharmacologic blockade of the MR in male mice prevented the preference for the unfamiliar mouse over the object. GR blockade had no effect. We conclude that the MR is required to fine-tune social abilities, especially in male mice.

### Increased sociability is regulated by mineralocorticoid receptor (MR)-mediated corticosterone action

The MR is involved in appraisal of novel situations. In an object exploration test, MR^CaMKCre^ mice showed an increased reactivity towards a novel vs. familiar object compared to controls (Berger et al., 2006). In this study, we offered the choice between two novel stimuli: a conspecific vs. an object, guided by the idea that MR might likely play a role in inhibitory control of social behavior but also in decision-making. Indeed, deletion of MR from the limbic circuitry of male and female mice even intensified the interest for their conspecifics.

To date, research on social behavior in rats demonstrated the involvement of the MR in territorial aggression and aggressive behavior induced by hypothalamic stimulation (Haller et al., 1998, 2000; Kruk et al., 2013; Ruiz-Aizpurua et al., 2013). Rats were adrenalectomized (removal of the adrenals and thus, the source of corticosterone) and supplemented with corticosterone to selectively activate MR. As a consequence of hormone suppletion the aggressive behavior became more violent. The most recent finding of these studies is that blockade of MR during the first aggressive encounter of rats, prevented the propensity of attack facilitation in subsequent conflicts in that same environment (Kruk et al., 2013). These studies clearly revealed the role of MR in violent aggression. We wished to fine-tune the role of MR in other more general aspects of social behavior: sociability (social interest) and social discriminative abilities.

The social approach test allows social interaction without aggressive attacks (Brodkin et al., 2004; Nadler et al., 2004; Moy et al., 2007; Smit-Rigter et al., 2010). In general, novel stimuli alive or not, trigger interest to be explored. When presenting two distinct novel stimuli to choose from, namely a conspecific in a cage and an empty cage (object), rodents decide and prefer to explore the rodent in the cage over the object (File and Hyde, 1978). In this task the test mouse is able to decide to go into a chamber with the stranger but is not forced to; it can leave and explore two other chambers if it wants to. This behavior signifies the relevance of decision-making influenced by social stimuli. To be adaptive, behavior towards both stimuli has to be balanced to respond correctly which requires inhibitory control mechanisms but also behavioral flexibility.

In this study we showed that male and female MR^CaMKCre^ mice explored the unfamiliar mouse much more than control mice did. We observed increased perseveration (visiting the same holes repeatedly) of male and female MR^CaMKCre^ mice on the spatial task of the circular hole board (ter Horst et al., 2012b, 2013b). Furthermore, MR^CaMKCre^ mice made more re-entry errors on the radial arm maze, especially female mice (Berger et al., 2006). Moreover, stressed female MR^CaMKCre^ mice showed high emotional arousal compared to their control littermates (Brinks et al., 2009). In addition, female MR^CaMKCre^ could not extinguish fear memory whereas male MR^CaMKCre^ could (Brinks et al., 2009; ter Horst et al., 2012a). All these findings point to a lack of behavioral flexibility in the absence of functional MR.

Whereas the GR is found throughout the brain the MR is most profoundly expressed in the hippocampus, lateral septum and amygdala regions (Reul and de Kloet, 1985; Ahima et al., 1991). With absence of MR and the concurrent imbalance of MR-GR in the hippocampus, one might expect a shift in inhibitory control of behavior (Gray and McNaughton, 2000). Gross hippocampal lesions and pharmacological treatment affecting the activity of the hippocampus are known to increase locomotion (Chan et al., 2001; Bast and Feldon, 2003; mice: Naert et al., 2013; rats: Sams-Dodd et al., 1997; Bannerman et al., 2001). Increased locomotion was seen in male MR^CaMKCre^ mice compared to the male controls indicating a lack of inhibitory control. When these MR-deficient mice were tested in an empty open field identical levels of activity were reported compared to their control littermates (Berger et al., 2006). This differential behavior of the male MR^CaMKCre^ mice in a stimulus-rich environment (the sociability cage) and a stimulus-poor environment (the open field) points to context-dependent behavior, which is a characteristic feature of the action of corticosteroid hormones (Schwabe et al., 2009).

Indeed, there seems to be a limbic control over behavioral inhibition via projections from the ventral hippocampus to the prefrontal cortex (PFC; Chudasama et al., 2012). Rats with ventral hippocampal lesions were not able to inhibit the impulsive urge to make a response in the 5-choice task, which is known to require the activation of the prefrontal cortex (Abela et al., 2013). Unfortunately, to our knowledge so far lesion studies are only performed in male rodents and not in females. So there could be a sex-specific effect on increased locomotion and this might explain why we did not see the hyperactivity in female MR^CaMKCre^ mice. In the sociability test, both male and female MR^CaMKCre^ mice showed increased interest in the stranger compared to the empty cylinder, even more than controls did. Interestingly, in a novel object recognition task MR^CaMKCre^ mice also persistently explored the novel object more than the controls (Berger et al., 2006).

To summarize, the MR is expressed in limbic brain regions but mainly in the hippocampus. In a human study it was found that the MR plays a role in inhibitory control (Schwabe et al., 2013). The limbic circuitry is also involved in inhibitory control with projections from the ventral hippocampus to the PFC. With the MR^CaMKCre^ mice showing impaired inhibitory control it seems likely that the hippocampus is the key in the neurocircuitry underlying decision-making.

### Sex differences in social discrimination

During the social discrimination test, we presented a novel mouse (new stranger) and the familiar mouse (old stranger). The natural tendency of rodents is to investigate an unfamiliar conspecific more than a familiar conspecific (Choleris et al., 2009); this is essential for male subjects in territorial defense which leads to an active exclusion of strangers. Male MR^CaMKCre^ mice did not show the expected stronger interest in the unfamiliar mouse compared to the familiar mouse as male control mice did. C57BL/6J, the background strain of the MR^CaMKCre^ mice, also preferred the company of the new stranger (Moy et al., 2007; Eagle et al., 2013).

Social discrimination in rodents is mainly based on olfactory cues (Young, 2002). During the social approach the olfactory bulb sends projections to the amygdala (Scalia and Winans, 1975), which is involved in a variety of social behavior, especially in processing social information (Ferguson et al., 2001). Because male MR^CaMKCre^ mice did not distinguish between the old and new stranger we had run a pilot study to test their olfactory ability in a buried pellet test. All mice found the pellet suggesting that these mice can smell, at least food; a deficit in processing olfactory signals seems to be unlikely. This observation is of interest because olfactory nuclei also express MR (Ahima et al., 1991); we do not know however if these MR were also deleted in our mutants.

The lack of discriminatory ability is sex-specific, because female MR^CaMKCre^ mice (and their controls) were more interested in the new stranger compared to the old stranger. Female MR^CaMKCre^ mice spent even more time with the new stranger than female control littermates. Again this points towards a lack of inhibitory control, but just for the males. To be adaptive, males should redirect their attention to the new stranger. To date, several studies showed that loss of MR in the forebrain enhanced sex differences in a context-dependent way, in cognitive and emotional behaviors (Berger et al., 2006; Brinks et al., 2009; ter Horst et al., 2012a,b, 2013a).

### Mineralocorticoid receptor (MR) rather than glucocorticoid receptor (GR) blockade influenced sociability

The balance in actions orchestrated by MR and GR is thought to be critical for stress responsiveness and behavioral adaptation (de Kloet et al., 1998, 2005). In response to the loss of MR in the hippocampus of MR^CaMKCre^ mice, an increase in GR expression was found in the CA3 region of the hippocampus and the mossy fiber tract (Berger et al., 2006; ter Horst et al., 2012b). To identify the receptor involved in the differences in social behavior in the male MR^CaMCKre^ mice we pharmacologically blocked the MR or the GR in control littermates and tested their sociability. In line with our expectations, GR blockade did not affect the exploration of the stranger: GR is responsible for consolidation of newly learned information (Oitzl and de Kloet, 1992; Roozendaal et al., 1996; Lupien and Mcewen, 1997; Oitzl et al., 2010) and would affect long-term behavior. Mice treated with the MR antagonist RU28318 spent the same amount of time with the conspecific and the object. This finding underlined that the MR is involved in social discrimination. The implication of the MR blockade was confirmed: one week later in a drug free state, these mice could indeed discriminate between an unfamiliar mouse and the object, spending more time exploring the unfamiliar conspecific. While MR antagonist treatment and genetic deletion both demonstrate that MR is implicated in sociability and discrimination, the actual outcome of both procedures is opposite. This could be related to the difference in phenotype produced by acute pharmacological blockade of the receptor vs. the conditional nature of the genetic deletion in early postnatal life implying lifelong adaptations to the lack of limbic MR.

### A role for mineralocorticoid receptor (MR) in inhibitory control

So far, the MR is known to be implicated in the appraisal and risk assessment of novel situations (Oitzl and de Kloet, 1992; Lupien and Mcewen, 1997) indicating its role in the early part of memory formation. A fast non-genomic action of corticosterone mediated by a membrane MR in the amygdala and hippocampus could be responsible for this (Karst et al., 2005, 2010; Groeneweg et al., 2011). Recently, in a stop-signal task which requires inhibition of the response to a signal it was found that acute stress increased inhibition in humans whereas this inhibitory enhancement was eliminated by a MR antagonist (Schwabe et al., 2013). This finding points towards an involvement of MR in stress-induced inhibitory control. In support of this suggestion, spironolactone, a MR antagonist, significantly impaired selective attention in men (Cornelisse et al., 2011). Spironolactone also significantly impaired selective attention and delayed recall of visuospatial memory and diminished set shifting/mental flexibility on a trend level in humans (Otte et al., 2007). These data are in support of our evidence from a social approach paradigm that limbic deletion of MR disinhibits a mechanism of sociability between conspecifics, particularly in females.

## Conclusion

The genetic deletion of MR from the limbic circuitry, especially the hippocampus, results in both sexes in an increased interest in an unfamiliar conspecific in both male and female mice, which can be viewed as a demonstration of impaired inhibitory control. Furthermore, unlike their control littermates male MR^CaMKCre^ mice could not discriminate between a familiar conspecific and an unfamiliar mouse whereas MR^CaMKCre^ females could. Since the MR^CaMKCre^ mice show impaired inhibitory control and behavioral flexibility we suggest that the hippocampus is a putative key region in the neurocircuitry involved in internal inhibition underlying decision-making in social behavior. Pharmacological blockade with a MR antagonist supported the evidence that the MR is needed to distinguish between a stranger and an object. Combined, these results suggest that in social behavior of particularly males the MR is needed to inhibit over-responding and to allow behavioral flexibility to differentiate between two conspecifics. The latter is especially important for survival in their natural environment.

## Conflict of interest statement

The authors declare that the research was conducted in the absence of any commercial or financial relationships that could be construed as a potential conflict of interest.
